# Prognostic Significance of B-Cells and pSTAT3 in Patients with Ovarian Cancer

**DOI:** 10.1371/journal.pone.0054029

**Published:** 2013-01-10

**Authors:** Chunmei Yang, Heehyoung Lee, Veronica Jove, Jiehui Deng, Wang Zhang, Xueli Liu, Stephen Forman, Thanh H. Dellinger, Mark Wakabayashi, Hua Yu, Sumanta Pal

**Affiliations:** 1 Department of Cancer Immunotherapeutics and Tumor Immunology, Beckman Research Institute, City of Hope Comprehensive Cancer Center, Duarte, California, United States of America; 2 Department of Biostatistics, City of Hope Comprehensive Cancer Center, Duarte, California, United States of America; 3 Department of Hematology, City of Hope Comprehensive Cancer Center, Duarte, California, United States of America; 4 Department of Surgery, City of Hope Comprehensive Cancer Center, Duarte, California, United States of America; 5 Department of Medical Oncology, City of Hope Comprehensive Cancer Center, Duarte, California, United States of America; Kinghorn Cancer Centre, Garvan Institute of Medical Research, Australia

## Abstract

**Background:**

Several previous studies have identified a strong association between T-cell infiltration and clinical outcome in ovarian cancer. The role of B-cells remains controversial, however.

**Methods:**

Forty-nine paraffin-embedded omental specimens derived from patients with high grade epithelial ovarian cancer were assessed. Immunohistochemical analyses were performed to characterize expression of CD19^+^ B-cells and pSTAT3 as high (>50% positively staining cells [PSCs]) or low (<50% PSCs). The Kaplan-Meier method with log-rank test was used to determine the association between clinicopathologic parameters and overall survival (OS). A multi-variate Cox proportional hazards regression analysis including nature of debulking (primary *vs* secondary), histology, tumor grade, receipt of prior chemotherapy, B-cell infiltration and pSTAT3 expression was performed.

**Results:**

Median OS was 160.6 months in those patients with low B-cell expression *vs* 47.3 months in those with high B-cell expression (P = 0.0015). Similarly, median OS was improved in those patients with low pSTAT3 expression (160.6 *vs* 47.9 months, P = 0.02). In a multivariate model to predict survival, only the degree of B-cell infiltration and clinical stage were retained. pSTAT3 expression did not enter the final model, possibly be due to a high positive correlation with B-cell infiltration (r = 0.82, P<0.0001).

**Conclusions:**

Increased B-cell infiltration and pSTAT3 expression in omental tissue are associated with poorer survival.

## Introduction

An amassing body of literature supports the role of immune cells in ovarian cancer proliferation. In 2003, Zhang *et al* reported a seminal study in which tissue from 186 patients was assessed for the degree of CD3^+^ tumor-infiltrating T-cells. [1] The presence of these cells was found to independently predict delayed recurrence and prolonged survival, with a large magnitude of effect. Several other studies have yielded the same conclusion, summarized recently in a meta-analysis by Hwang *et al.* [2] With a cumulative experience of 1,815 patients across 10 studies, it was demonstrated that a lack of tumor-infiltrating lymphocytes (TILs) was associated with poorer survival (hazard ratio [HR] 2.24; 95% CI 1.71–2.91). In these studies, TILs were characterized by expression of CD3 and/or CD8. [1], [3], [4], [5], [6], [7], [8], [9], [10], [11] The observed correlations between T-cell infiltration and clinical outcome is a biologically plausible one – specifically, CD8^+^ T-cells may cause a direct cytolytic antitumor effect.

With a greater understanding of T-cell biology, it has become clear that certain subsets of T-cells may actually have a procancer effect. Specifically, Barnett *et al* have assessed the extent of regulatory T-cell (Treg) infiltration in a series of 232 primary serous ovarian cancer specimens. [12] In this series, the extent of Treg infiltration was associated with higher cancer grade and advanced clinical stage. Corroborating this data, peripheral blood assessments from patients with ovarian cancer suggest and increasing number of circulating Tregs accompanying disease progression. [13]

The role of B-cells in ovarian cancer is more challenging to discern. [14] In early studies using murine models of B-cell deficiency, it was suggested that B-cells may actually inhibit the antitumor effect of tumor infiltrating T-cells. [15], [16] As a potential mechanism, B-cells have been demonstrated to inhibit the priming effect of CD4^+^ T-cells on CD8^+^ T-cells. [17] Despite these observations, clinical evidence points to a link between CD20^+^ tumor infiltrating B-cells and a favorable outcome in the setting of ovarian cancer. Specifically, Milne *et al* examined tissue microarray (TMA) data derived from 199 patients with high-grade serous epithelial ovarian cancer (EOC). [18] In this report, the presence of intraepithelial CD20+ cells was associated with an improvement in disease-specific survival (DSS). However, it is important to note that this report was accompanied by other paradoxical observations – for instance, the presence of FoxP3^+^ regulatory T-cells (Tregs) were also associated with a favorable DSS. This finding contradicts the preponderance of evidence suggesting that Tregs may have a pro-cancer effect.

Clearly, the relationship between B-cells and clinical outcome warrants further assessment. In the current manuscript, we assess the extent of B-cell infiltration in omental tissue derived from patients with ovarian cancer, and attempt to correlate the extent of infiltration with overall survival (OS). We have previously demonstrated that phosphorylated signal transducer and activator of transcription-3 (pSTAT3) plays a critical role in cancer-related inflammation and tumor progression through recruitment of myeloid cells and Tregs. [19], [20] More recently, we have demonstrated a critical role of STAT3 in promoting B-cell mediated tumor angiogenesis in mouse models (unpublished data). To this end, we further explored the association of between pSTAT3 and B-cell infiltration.

## Methods

### Ethics Statement

Clinical and pathologic data assessed in this study were anonymized, and the research on clinical specimens was approved by the City of Hope IRB (COH IRB 10072).

### Study Population

The City of Hope Biospecimen Repository (COHBR) houses tissue derived from all surgical procedures performed at the institution. Through an IRB-approved protocol, we obtained paraffin-embedded sections of omental tissue from patients who possessed a diagnosis of high-grade EOC (i.e., grade 2 or 3) and had primary or secondary debulking performed at the institution. Specimens were derived from debulking procedures performed between January of 2000 and December of 2009, and sufficient sample had to be available to generate a total of 12 unstained slides (4 µm thickness). For patients receiving primary debulking, use or nonuse of prior (i.e., neoadjuvant) chemotherapy was characterized. For patients receiving secondary debulking, the use of prior chemotherapy included adjuvant chemotherapy rendered after primary debulking. Survival was characterized from the time of diagnosis with ovarian cancer (importantly, not from the time of primary or secondary debulking surgery).

### Tissue Analyses

Paraffin tissue slides were de-paraffinized in xylene, re-hydrated through graded alcohols, and subsequently autoclaved in Antigen Unmasking Solution (Vector Laboratories). For IF staining, tissue sections were incubated with primary antibody in a dilution of 1∶50. The slides were then incubated in fluorophore-conjugated secondary antibody. Images were taken by confocal microscopy using CLSM 510 Meta confocal microscope (Zeiss). For IHC staining, tissue sections were treated with 1% H_2_O_2_ in methanol for 10 min at room temperature, and then incubated for one hour in PBS containing 10% goat serum (Sigma). Sections were incubated overnight at 4°C with primary antibody. After incubation with biotinylated secondary antibodies, ABC/DAB detection method was performed according to the manufacture’s instructions (Vector Laboratories). Tissue sections were subsequently counterstained with hematoxylin for 30 sec. The expression level of primary antibody in tumor tissues was visualized by a Nikon ECLIPSE TE2000-U microscope (4× objective magnification) and imaged using SPOT software. The primary antibodies used are anti-pY705-STAT3 (Cell Signaling) and anti-CD19, a marker for human B-cells (AbD Serotec). In all cases, ten microscopic fields with at least 1,000 cells were counted per each slide for scoring. Scoring result from IHC-stained tissue sections were further confirmed by IF staining in a consecutive section.

### Statistical Analysis

A semi-quantitative scale was used to assess the extent of CD19 and pSTAT3 staining. Specifically, those specimens with 50% of cells with positive staining (or greater) were considered “high”, and those specimens with less were considered “low”. These scores were assigned by independently by two investigators (C.Y. and H.L.), and discrepant scores were then arbitrated to yield a uniform assignment. The Kaplan-Meier method was used to compare survival in cohorts defined by (1) primary or secondary debulking, (2) B-cell staining (high *vs* low), (3) pSTAT3 staining (high *vs* low), and a combination of these parameters. A multi-variate Cox proportional hazards regression analysis was conducted to investigate whether B-cells or pSTAT3 staining provides additional predictive power in survival beyond nature of debulking (primary *vs* secondary), histology (serous, mucinous, or endometrioid *vs* clear-cell or undifferentiated), tumor grade (2 *vs* 3), or prior chemotherapy (yes *vs* no). All statistical analyses carried out using R software (available at http://www.r-project.org/).

## Results

### Patient Characteristics

**Figure 1:**
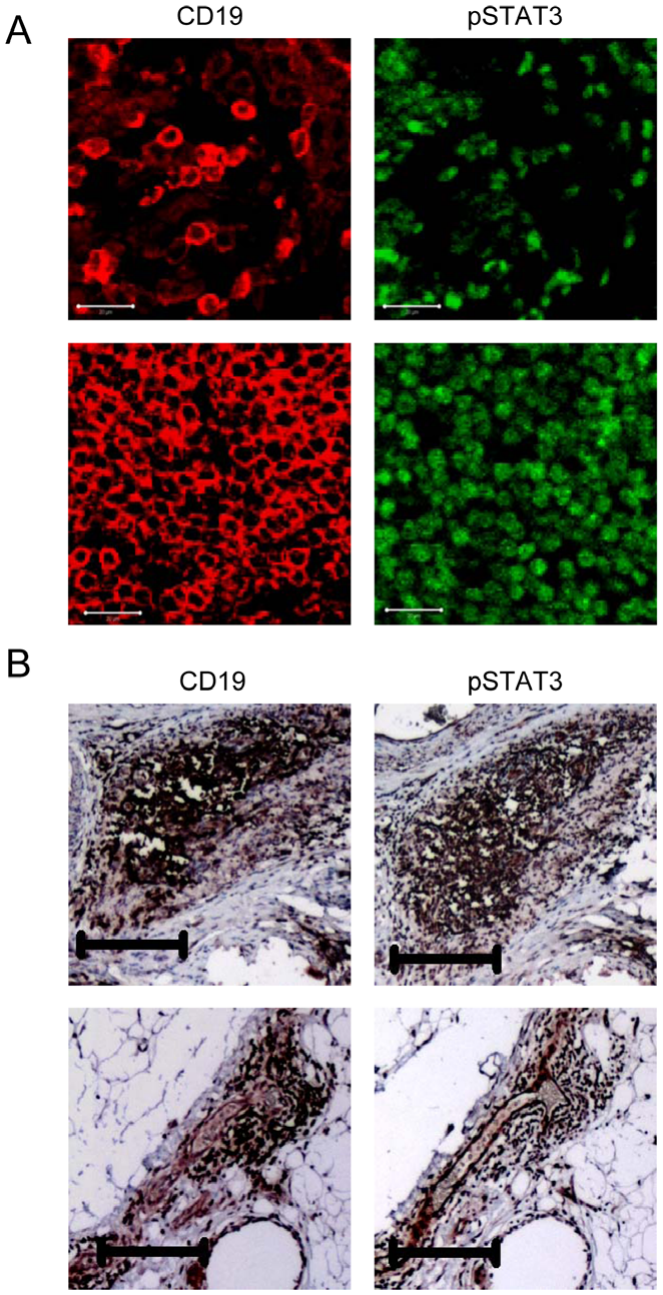
pSTAT3-positive B cells are readily detectable in the omental tissues of ovarian cancer patients. (**A**) Immunofluorescent staining followed by confocal microscopy showing examples of representative specimens from a patient with low B-cell infiltration and low pSTAT3 expression (top left and right), and a separate patient with high B-cell infiltration and high pSTAT3 expression (bottom left and right); scale bars, 20 µm. (**B**) IHC images showing B cells and pSTAT3-positive cells in the same area of omental tissues; scale bars, 200 µm.

From the COHBR, a total of 110 pathologic specimens yielded from debulking surgeries from ovarian cancer were identified. Of these, a total of 29 specimens represented histologies not relevant to the current study (i.e., low malignant potential tumors, carcinosarcomas, or germ cell tumors). Of the remaining 81 cases, 17 specimens represented grade 1 tumors, and 15 specimens did not have sufficient omental tissue for the proposed analyses, yielded a total of 49 specimens for the current study. The median age of the patients represented with the current cohort was 61 years (range, 41–87), and the majority of patients were characterized as having serous, endometroid or mucinous histology (47 of 49, or 96%). Eighteen patients (37%) had grade 2 disease, while 31 patients (63%) had grade 3 disease. The majority of specimens were derived from primary debulking surgeries (30 of 49, or 61%), and the remainder from secondary debulking. Most patients who had undergone primary debulking had received no prior systemic treatment (3%), while the majority of patients who had undergone secondary debulking had received chemotherapy (89%). Table 1 presents clinical and pathologic characteristics subdivided the extent of B-cell expression. No significant differences were seen amongst groups with the exception of the extent of pSTAT3 staining – specifically, the preponderance of patients with high B-cell staining also demonstrated high pSTAT3 staining, while those with low B-cell staining demonstrated low pSTAT3 staining (P<0.0001). Examples of low and high B-cell infiltration and pSTAT3 expression are shown in Figure 1A. Moreover, B-cells and pSTAT3-positive cells were found in the same area of patient omental tissue (Figure 1B).

**Table 1:** Clinical and pathologic characteristics.

Characteristic		Low B-cells(N = 20)	High B-cells(N = 29)	P-value
Age – yr	Mean	62.6	60.3	0.53
	Range	41–87	43–80	
Histologic Subtype – no. (%)	Serous, mucinous, or endometrioid	18 (90%)	29 (100%)	0.16
	Clear-cell or undifferentiated	2 (10%)	0 (0%)	
Initial clinical stage– no. (%)	I-II	12 (60%)	4 (14%)	0.001
	III-IV	8 (40%)	25 (86%)	
Grade – no. (%)	2	8 (40%)	10 (34%)	0.77
	3	12 (60%)	19 (66%)	
Source of tissue	Primary debulking	12 (60%)	18 (62%)	1.00
	Secondary debulking	8 (40%	11 (38%)	
Primary chemotherapy prior to debulking – no. (%)	Yes	0 (0%)	1 (3%)	1.00
	No	12 (60%)	17(59%)	
Secondary chemotherapy prior to debulking – no. (%)	Yes	7 (35%)	10 (35%)	
	No	1 (5%)	1 (3%)	
STAT3 status – no. (%)	High	0 (0%)	25 (86%)	<0.0001
	Low	20 (100%)	4 (14%)	

### B-cell/pSTAT3 Expression and Clinical Outcome: Univariate Analysis

**Figure 2:**
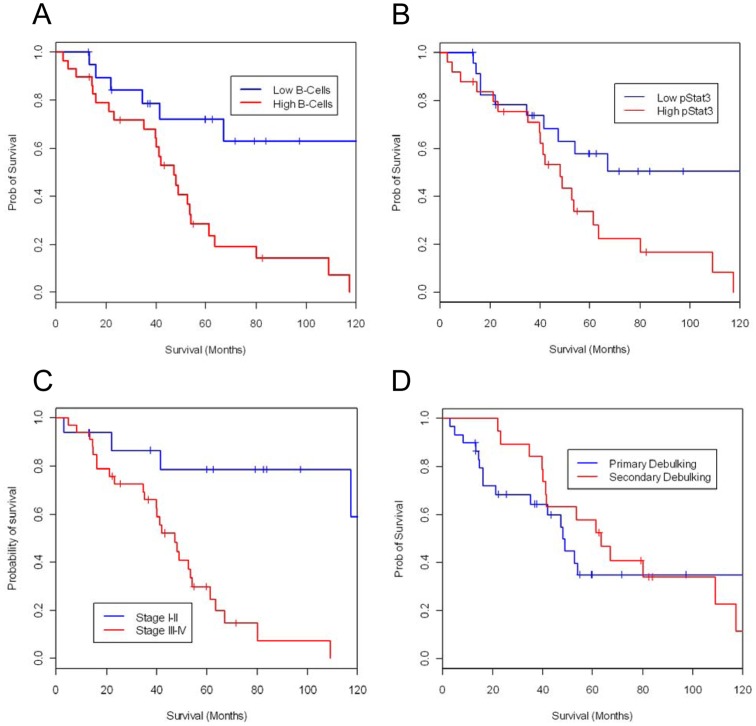
B cell clustering and pSTAT3 in the omental tissues predict poor prognosis in patients with ovarian cancer. Kaplan-Meier curve illustrating the duration of survival in patients with ovarian cancer based on (**A**) degree of B-cell infiltration (P = 0.0015), (**B**) degree of pSTAT3 expression (P = 0.02), (**C**) histological staging system and (**D**) debulking status.

Given the mixed population of patients examined in the current study (i.e., patients undergoing primary and secondary debulking procedures), OS was the principal endpoint examined. Median OS was 160.6 months in those patients with low B-cell expression as compared to 47.3 months in those with high B-cell expression (P = 0.0015; Figure 2A). Similarly, median OS was improved in those patients with low pSTAT3 expression as compared to high pSTAT3 expression (160.6 *vs* 47.9 months, P = 0.02; Figure 2B). Although there was an improved median OS in those patients with stage I/II disease *vs* stage III/IV disease (P = 0.001), nature of debulking surgery (primary *vs* secondary; P = 0.56) and tumor grade (i.e., 2 *vs* 3; P = 0.16) did not appear to have an impact on clinical outcome (Table 2).

**Table 2:** Results of univariate and multivariate analyses.

		Univariate		Multivariate	
		Hazard Ratio (95% CI)	p-value	Hazard Ratio (95% CI)	p-value
Stage*	I–II	1.00 (reference )			
	III–IV	7.60 (2.22–26.0)	0.001	5.16 (1.36–19.6)	0.016
Nature of Debulking	Primary	1.00 (reference )			
	Secondary	0.80 (0.39–1.67)	0.56	–	–
Histology	Serous, mucinous or endometrioid	1.00 (reference )			
	Clear cell or undifferentiated	0.75 (0.10–5.58)	0.78	–	–
Tumor Grade	2	1.00 (reference )			
	3	1.96 (0.82–4.71)	0.13	–	–
Prior chemotherapy	Yes	1.00 (reference )			
	No	1.07 (0.54–2.10)	0.85	–	–
B-cells*	Low	1.00 (reference )			
	High	3.93 (1.58–9.72)	0.003	2.03 (0.75–5.50)	0.16
pSTAT3	Low	1.00 (reference )			
	High	2.37 (1.10–5.09)	0.03	–	–

### Multivariate Analysis

Using a multivariate Cox proportional hazards regression analysis incorporating initial clinical stage (I–II *vs* III–IV), nature of debulking (primary *vs* secondary), histology (serous, mucinous, or endometrioid *vs* clear-cell or undifferentiated), tumor grade (2 *vs* 3), prior chemotherapy (yes *vs* no), B-cell expression (high *vs* low) and pSTAT3 expression (high *vs* low), only the degree of B-cell infiltration (hazard ratio, HR, 2.03, 95% CI 0.75–5.50) and stage (HR, 5.16, 95% CI 1.36–19.60) were retained in the multivariate model. Given this observation, we explored the correlation between stage and B-cell infiltration and found a modest correlation (r = 0.38, P<0.001). pSTAT3 did not enter the final model, which may possibly be due to its high positive correlation with B-cell expression (r = 0.82, P<0.0001) (Table 2).

## Discussion

Several unique observations emerge from the current study. A greater extent of B-cell infiltration in omental tissue appears to correlate with poorer survival. B-cell infiltration is associated strongly with pSTAT3 expression in omental tissue, and in fact, increased pSTAT3 expression in omental tissue is also associated with poorer survival. On multivariate analysis, B-cell infiltration entered the final multivariate model along with clinical stage, and a strong association was noted between pSTAT3 expression and B-cell infiltration.

As already noted, the impact of T-cell infiltration is relatively well documented in the setting of ovarian cancer, now summarized in a meta-analytic dataset. [2] These correlative studies have spurned efforts to employ adoptive transfer of T-cells, both alone and in combination with cytotoxic therapy. [21], [22], [23] The effects of relevant T-cell subsets, such as the procancer effects of Tregs, have also been discerned. The role of B-cells remains more controversial – although traditionally thought to have an antagonistic effect on cytotoxic T-cell responses, the aforementioned dataset from Milne *et al* suggests that tumor infiltrating B-cells may be a positive prognostic marker. However, it is important to note that our study utilized a distinct B-cell marker (CD19), and also assessed a more heterogeneous group of patients (patients who received secondary debulking and prior systemic therapy). With respect to the latter, we found no significant difference in outcome based on these clinical features, and surprisingly, the distribution of biomarkers did not vary in these subgroups. Our study also assessed individual sections derived from paraffin-embedded specimens; in contrast to other studies evaluating TMAs, this approach allowed for assessment of multiple fields (10 per specimen) to provide a more accurate biomarker assessment.

The close association between B-cells and pSTAT3 in this study supports preclinical data we have previously reported delineating the role of pSTAT3 in immune cell recruitment. [24] Our preclinical data also suggest that abrogation of STAT3 activity may have an antitumor effect, consistent with our clinical observation that higher pSTAT3 may be associated with poorer clinical outcome. [25], [26] In combination, if these clinical observations are confirmed in larger series, this may serve as rationale to pursue therapeutic trials of STAT3 antagonists to target B cells in ovarian cancer. As one example, we have developed a CpG-conjugated siRNA directed at STAT3 in myeloid cells and B cells. [26] While the delivery of siRNA-based therapies has been a formidable challenge, ovarian cancer may be an ideal disease entity to approach with these agents. Intraperitoneal chemotherapy has a well-established survival benefit in phase III studies, and intraperitoneal delivery of RNA-based therapeutics may circumvent certain stability and solubility issues. [27] In addition to directly antagonizing STAT3, an approach targeting upstream moieties such as JAK2 could be considered. Several JAK2 inhibitors have been developed for the treatment of myeloproliferative diseases bearing aberrations in this protein; it has been demonstrated that one of these small molecules, AZD1480, has an antitumor effect in the MDAH2774 ovarian cancer cell line. [28], [29].

Several limitations should be acknowledged. Firstly, our sample size was relatively small in contrast to other published reports. [1], [30] Secondly, we did not assess T-cell populations (i.e., CD8^+^ T-cells and FOXP3^+^ regulatory T-cells) to confirm previously reported findings. [30] Third, it remains unclear what effect certain treatment variables (i.e., prior chemotherapy) may have had on the observed B-cell or pSTAT3 distribution – this warrants further study in a larger series. Finally, our study exclusively focuses on tissue-based markers. Although conventional serum markers such as the CA-125 have fallen out of favor after recent prospective assessments, there are a multitude of new serum-based assays on the horizon. [31] For instance, autoantibody profiling studies have identified signatures that differ between ovarian cancer patients and healthy controls. [32] It will be curious to see whether a combination of the biomarkers we have proposed herein with others may yield superior personalized prognostic tools. Despite these limitations, our data adds to a very limited body of literature related to the impact of B-cell infiltration on clinical outcome in advanced ovarian cancer, and provides a plausible mechanism for this to occur (namely, B cell recruitment via STAT3-dependent pathways). Clearly, larger efforts will be needed to validate the observations made herein. With a shadow cast over traditional biomarkers such as the CA-125, immune markers hold immense promise in ovarian cancer. [33] Given a burgeoning pipeline of JAK/STAT pathway inhibitors, our data linking pSTAT3 expression and B-cell infiltration to clinical outcome may ultimately support a novel therapeutic avenue for this disease.
